# Association of Chrono-Nutritional Profiles with Weight Loss and Comorbidity Remission After Bariatric Surgery in Patients with Severe Obesity

**DOI:** 10.3390/nu17172901

**Published:** 2025-09-08

**Authors:** Silvia Bettini, Enrico Carraro, Anna Pilatone, Sami Schiff, Paolo Girardi, Matteo D’Angelo, Anxhela Begolli, Fatemeh Mansouri, Saba Toosinezhad, Sara Sandri, Beatrice Gusella, Gabriella Milan, Mirto Foletto, Paola Fioretto, Luca Busetto

**Affiliations:** 1 Centre for the Study and the Integrated Treatment of Obesity, Department of Medicine, Padova University Hospital, 35128 Padova, Italy; d.ssa.silvia.bettini@gmail.com (S.B.); fatemeh.mansouri@unicam.it (F.M.);; 2 Department of Statistical Sciences, University of Padova, 35121 Padova, Italy; 3 Department of Environmental Sciences, Informatics and Statistics, Ca’ Foscari University of Venice, 30172 Venezia, Italy

**Keywords:** obesity, chrono-nutrition, psychological parameters, physical and mental health, bariatric surgery

## Abstract

Background/Objectives: A disruption of eating habits is related to obesity and obesity-related complications (ORCs), including diabetes and cardiovascular disease. We previously described chrono-nutritional profiles considering the eating habits of patients with severe obesity during the 24 h cycle. Our present study aims to determine, first, whether belonging to a specific eating profile is associated with greater or lesser weight loss in patients with obesity who have undergone bariatric surgery, and second, whether chrono-nutritional profiles are associated with the remission of ORCs after surgery. We also investigated whether there were differences between the original baseline profile and the new profile derived from the 24 h recall on dietary patterns. Methods: The study included 75 patients with obesity who had undergone bariatric surgery and were followed up for a period of 36 ± 11 months. Data were collected from patients’ medical records and telephone interviews. R software (v4.4.0; R Core Team, 2024) was used. Results: Significant weight loss from baseline was observed at follow-up for all profiles (*p* < 0.0001); however, there were no significant differences in weight loss % among profiles. Using a linear regression model, Profile 3 (characterized by irregular eating patterns) demonstrated less weight loss at follow-up compared to other profiles (*p* = 0.0487). There was a significant remission of ORCs from baseline to follow-up, but there were no significant differences among profiles. Conclusions: Chrono-nutritional profiles may play a role in weight regulation in patients with severe obesity who are candidates for bariatric surgery. Further studies with a larger sample size are needed.

## 1. Introduction

By 2035, it is estimated that 1 in 4 people in the world, approximately 1.9 billion, will be affected by obesity, with childhood obesity expected to increase by 100% [1,2]. Obesity is a chronic, relapsing, non-communicable disease characterized by an excessive and pathological accumulation of body fat [3]; it is also defined by the presence or absence of functional alterations in organs and tissues [4]. It is well established that obesity acts as a trigger for a wide range of other non-communicable diseases, known as obesity-related complications (ORCs), including diabetes and cardiovascular disease. Obesity is caused by multiple factors, among which a chronic positive energy balance is one of the main drivers [5,6]. Therefore, dietary modification, particularly the control of eating behaviours, is an essential element in the management of obesity. Chrono-nutrition is an emerging area of research that studies the interaction between circadian rhythms, temporal eating patterns, and metabolic health [7,8,9]. The circadian clock regulates daily energy metabolism and daily rhythms of sleep/wake, fasting/feeding, and catabolic/anabolic cycles, body temperature, and endocrine functions. The human circadian system operates on an approximately 24 h cycle, but lifestyle and environmental factors, such as shift work and access to energy-dense food, can disrupt this system and thereby adversely affect individual health. Growing evidence demonstrates a complex reciprocal relationship between metabolism and the circadian system, in which perturbations in one system affect the other [10,11]. Irregular, random, and ad libitum eating increases the probability of the consumption of high-energy foods. Other studies have suggested that reduced eating frequencies can negatively impact appetite control, and long periods of fasting or skipping meals can lead to larger portion sizes due to compensatory eating [10,12]. The timing of food intake has been reported as essential in maintaining metabolic health or treating impaired metabolic processes in pathological conditions [13,14]. Furthermore, intake in the morning is more satiating and may reduce the total amount of caloric intake for the day, and intake late at night may result in greater overall daily intake [15]. It was demonstrated that the evening chronotype, characterized by the consumption of most food in the last hours of the day, is associated with a higher intake of energy drinks and fat, suggesting a link with cardiometabolic health and obesity [16,17].

The evening chronotype has also been correlated with increased risk of type 2 diabetes, gastrointestinal disorders, and psychiatric symptoms compared to the morning chronotype [18]; it is also correlated with an increased risk of all-cause mortality over 6.5 years [19]. Conversely, those who consume their largest meal at breakfast rather than dinner had a significant decrease in their Body Mass Index (BMI) in a 7-year follow-up [20]. Furthermore, late lunch eaters seem to lose less weight than early lunch eaters, independent of energy intake or dietary composition [21]. In healthy individuals, eating earlier in the day improves weight and several key metabolic outcomes [22].

Similarly, in women with overweight and obesity high caloric intake during breakfast compared to high caloric intake at dinner was shown to lead to greater weight loss and waist circumference reduction [23]. A randomized control trial also demonstrated that high energy intake at breakfast is associated with a significant reduction in overall postprandial hyperglycaemia in patients with type 2 diabetes [24].

Restricting the timing of food availability (i.e., time-restricted feeding or time-restricted eating (TRE)) has been shown to benefit the circadian system and metabolic functioning in humans. Increasing the time of fasting can delay and often reverse metabolic disorders, reducing insulin resistance and increasing glucose tolerance [25]. Particularly, fasting for 18 h can induce a switch from glucose-based to ketone-based metabolism, which leads to increased longevity and a decreased incidence of diseases, including obesity [26]. It was also demonstrated that early TRE reduces appetite and increases metabolic flexibility, which is the ability to switch between carbohydrate and fat oxidation, without affecting energy expenditure [27].

Moreover, studies on the timing of eating demonstrated not only a positive effect on weight loss, but also an improvement in metabolic-related disease in humans.

In our previous study, we described chrono-nutritional profiles based on an analysis of the eating habits of patients with severe obesity during the 24 h cycle and investigated potential relationships between these profiles, medical comorbidities, and psychological status [28]. In the present follow-up study, we aimed to determine how different eating patterns in patients with obesity who underwent bariatric surgery affected their weight loss and remission from comorbidities three years after surgery.

## 2. Materials and Methods

### 2.1. Patient Multi-Disciplinary Assessment

In this observational retrospective study, 193 patients with severe obesity qualified for bariatric surgery according to the European guidelines for obesity management in adults [29] were enrolled in the Centre for the Study and the Integrated Treatment of Obesity at the University Hospital of Padua, Italy. An extensive multi-disciplinary assessment was performed, including medical assessment, biochemical testing, psychological evaluation with psychometric tests, and dietetic assessment, as previously shown [28]. All patients received nutritional education and counselling. In the first study, 173 patients with complete information on all the domains of the multi-disciplinary evaluation (medical, psychological, psychometric, and dietetic assessment with biochemical testing) were recruited.

Among the 173 patients in the first study, the patients who qualified for this follow-up study met the criteria of undergoing bariatric surgery and attending the follow-up visits at 3 years (36 ± 11 months). Based on these criteria, we recruited 75 patients. All subjects gave consent for the use of the data. The protocol was approved by the Ethics Committee of Padua for Clinical Research (Study nr:480n/A0/24).

The data was collected from the patients’ medical records from the hospital database and telephone interviews. Patients were asked questions about their eating patterns (such as the number of meals they consume), alcohol consumption and smoking habits, presence/absence of comorbidities, drug intake, and their sleeping schedule.

The variables analyzed are classified into baseline and follow-up variables. The baseline variables included sex, age, anthropometric parameters, education, smoking and alcohol habits, physical activity, biochemical assessment, and presence of ORCs (hypertension, prediabetes, diabetes, Obstructive Sleep Apnea (OSA), and dyslipidemia). The follow-up variables were sex, age, anthropometric parameters, smoking and alcohol habits, sleeping schedule, physical activity, presence of comorbidities (hypertension, prediabetes, diabetes, OSA, and dyslipidemia), and eventual presence of Major Adverse Cardiovascular Events (MACE). To evaluate physical activity at follow-up, we used the Global Physical Activity Questionnaire (GPAQ). The GPAQ covers several components of physical activity, such as intensity, duration, and frequency, and it assesses three domains in which physical activity is performed (occupational physical activity, transport-related physical activity, and physical activity during discretionary or leisure time) [30]. The Epworth Sleepiness Scale (ESS) was used to assess daytime sleepiness in our patients. Patients were asked how likely they are to doze off or fall asleep on a scale of 0–3, in 8 specific situations, with total scores ranging from 0 to 24. An ESS score of 10 or greater is considered indicative of subjective excessive daytime sleepiness [31].

Weight loss % was calculated as follows:

Weight loss (%) = (Baseline weight (kg) − Weight at follow-up (kg))/ (Baseline weight (kg)) × 100

### 2.2. Chrono-Nutritional Profiling

As shown in our previous study [28], we identified 4 chrono-nutritional profiles based on the likelihood of food contact at different times throughout the 24 h cycle in 173 patients with severe obesity. Clusters of patients with a similar pattern of daily nutrition were created using the 24 h dietary recall. The strategy used consists of matching each meal with the relative time of consumption. Subjects in Profile 1 reported having mostly three daily meals (“meal eaters”). Patients in Profile 2 had three main meals and a couple of snacks in between (“meal and snack eaters)”. Profile 3 consisted of patients who reported having many different kinds of meals, including nibbling, predominantly in the daytime but with a certain percentage of eating during the night (“continuous day and night eaters”). Profile 4 had the same pattern as the previous one, but without nibbling occurring during the night (“continuous day eaters”). At the FU evaluation, telephone interviews were conducted to collect information about the eating patterns of the 75 patients, including the number of meals they consume, the timing of food intake, and meal type, based on 24-h dietary recalls, with the aim of reclassifying patients according to their new eating profiles.

Patients were grouped into four different clusters based on their eating pattern, initially based on their baseline profile, and later based on their profile 3 years after bariatric surgery. These profiles were compared in terms of weight loss, BMI, and comorbidities.

### 2.3. Statistical Analysis

Statistical analyses were performed using the software R (v4.4.0; R Core Team, 2024) [32].

Categorical variables were expressed as frequency (%), whereas quantitative variables were expressed as mean ± standard deviation (SD) if the dataset followed a normal distribution, or as median (interquartile range (IQR)) if the dataset did not follow a normal distribution. All variables were tested for normal distribution with the Shapiro–Wilk test. If the normality assumption was not met for two paired samples, the test used was the Wilcoxon Signed-Rank test. Statistical significance was assumed at the 5% level. Changes from the baseline profile to the follow-up profile were tested via Pearson’s Chi-squared test.

With the aim of evaluating which factors influenced the outcomes of interest, we performed a linear regression on the mean value, as it is important that the residuals are normally distributed, rather than the variable itself. Variables were selected using the AIC index and a backward approach starting from a full model, which included gender (male or female), age in continuous form, physical activity (GPAQ), current alcohol consumption (no or yes), smoking habits (never smoked, current smoker, or former smoker), presence of diabetes at baseline, and chrono-nutritional profile at baseline or follow-up. The model results were presented as coefficient estimates and their relative 95% Coefficient Intervals (95% CIs). Clinical variables such as diabetes were distributed on a binary scale (absence/presence), justifying the use of a logistic binary regression model. To estimate the parameters, we used the bias correction method proposed by Firth (1993) [33]. This regression model aimed to verify if belonging to a precise profile at baseline and follow-up determines the remission of a comorbidity. Variables were selected using the AIC index and a backward selection procedure starting from a full model, including the same variables used in the model based on the quantile regression. The results were presented using Odds Ratios (ORs) or an exponential transformation of the model coefficients with their relative 95% Coefficient Intervals (95% CIs).

## 3. Results

### 3.1. Characteristics of the Population According to Different Profiles at Baseline and Follow-Up, Before and After Bariatric Surgery

To study whether membership in a specific profile can be associated with different weight loss outcomes, we divided patients, first, according to their baseline profile, and second, according to their profile 3 years after bariatric surgery.

Among the 173 patients in the first study, only 75 patients who underwent bariatric surgery and attended the follow-up visits at 3 years (36 ± 11 months) were recruited. The sample showed a significant predominance of females, accounting for 74.67% of the population. The median baseline weight was 114 (IQR 104.50–131.50) kg, while the baseline BMI was 42.2 (IQR 39.10–46.35) kg/m^2^. At the follow-up, the median weight measured was 81 (IQR 73–93) kg and the median BMI was 29.48 (IQR 27.45–33.92) kg/m^2^ with a minimum of 20.8 kg/m^2^ and a maximum of 43.91 kg/m^2^ (Table 1 and Table 2). Patients were grouped in four different profiles based on their baseline eating profiles (Table 1). The most frequent profile was Profile 4 (n = 38, 28.5%), and the least frequent one was Profile 1 (n = 8, 6%). The profiles were compared for weight/BMI and the presence of ORCs before and after surgery, denoting no significant differences among all variables.

Patients were re-classified according to their follow-up profiles, and the characteristics of the population before and after surgery are reported in Table 2. The most frequent profiles are Profile 2 and Profile 4 (n = 33, 44%). Profile 1 consists of five patients (6.67%), and Profile 3 consists of four patients (5.33%). In contrast to the baseline profiles, a statistically significant difference in gender was observed, with a higher prevalence of males in Profile 1 (only 20% of females) (*p* = 0.0112).

**Figure 1:**
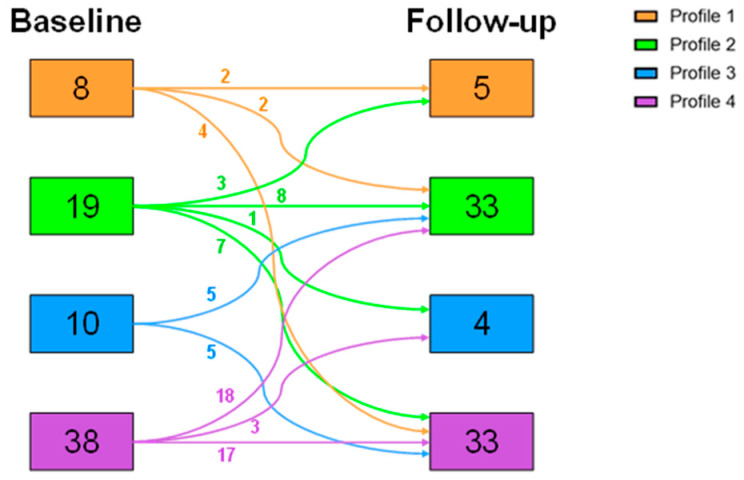
Changes in profiles before and after weight loss, expressed in number of patients. Profile 1: meal eaters; Profile 2: meal and snack eaters; Profile 3: continuous day and night eaters; Profile 4: continuous day eaters. Pearson’s Chi-squared test: *p* = 0.191.

The changes from the patients’ baseline profile to the follow-up profile were studied. As represented in Figure 1, the main difference was the increase in the patients belonging to Profile 2 (from 19 to 33 patients), while Profile 4 maintained a similar prevalence (from 38 to 33 patients). However, no significant differences in the distribution from the baseline profiles to the follow-up profiles were observed (Pearson’s Chi-squared test: *p* = 0.191).

**Figure 2:**
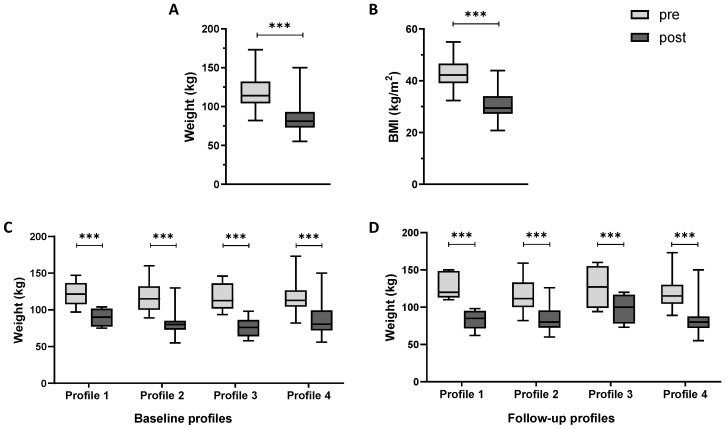
Weight (kg) (**A**) and body mass index (BMI) loss (kg/m^2^) (**B**) in all population before (pre, light grey box plot) and after (post, dark grey box plot) bariatric surgery. (**C**) Weight loss classifying patients according to their baseline profiles and (**D**) according to their follow-up profiles. Results were presented as a box plot, with 25th, 75th percentile and median values. Profile 1: meal eaters; Profile 2: meal and snack eaters; Profile 3: continuous day and night eaters; Profile 4: continuous day eaters. *** *p* < 0.0001.

Overall, patients showed significant weight (*p* < 0.0001) and BMI loss (*p* < 0.0001) 3 years after bariatric surgery (Table 1 and Table 2 and Figure 2A,B). Clustering patients according to their baseline chrono-nutritional profiles, we evidenced the same statistically significant decrease (*p* < 0.0001) (Figure 2C) in all profiles. In the same way, also considering the weight loss for each follow-up profile, the difference was statistically significant (*p* < 0.0001), as shown in Figure 2D.

**Figure 3:**
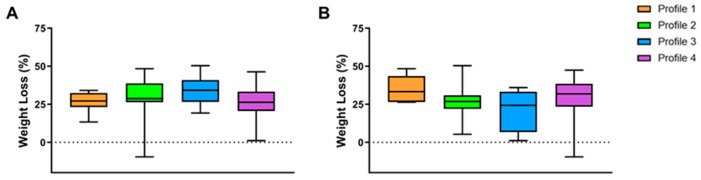
Weight loss % clustering patients according to their baseline profiles (**A**) and follow-up profiles (**B**). Results were presented as a box plot, with 25th, 75th percentile and median values. Profile 1: meal eaters; Profile 2: meal and snack eaters; Profile 3: continuous day and night eaters; Profile 4: continuous day eaters.

Considering the difference between weight loss % among the four different profiles, there were no significant differences considering both baseline profiles (*p* = 0.07) and follow-up profiles (*p* = 0.15) (Figure 3A,B).

**Figure 4:**
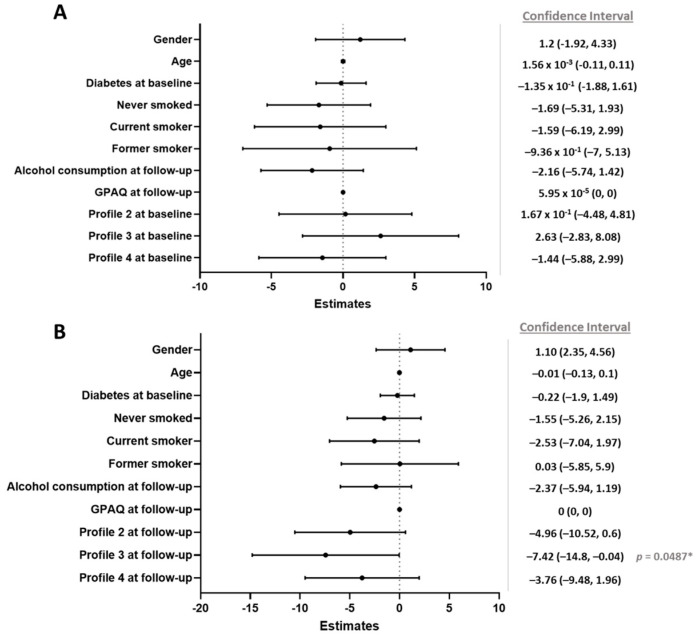
Forest plot with estimates to predict BMI loss using baseline profiles (**A**) and follow-up profiles (**B**), respectively. The interval represents the 95% confidence intervals estimated by a logistic regression, with respect to the reference category: gender [Female], alcohol consumption [No], smoking habit [No], and diabetes [No]. *: *p* < 0.05. Profile 2: meal and snack eaters; Profile 3: continuous day and night eaters; Profile 4: continuous day eaters; GPAQ: Global Physical Activity Questionnaire.

We built a linear regression model to analyze the effects of each variable on the outcome (BMI loss) to clarify which factors represented independent determinants of BMI loss after bariatric surgery. We decided to include the BMI in the regression model rather than weight because of its natural correlation with the definition of obesity and obesity classes. The variables included in the analyses because of their known correlation with weight modifications, and thus considered confounding variables, were gender, age, presence of diabetes at baseline, smoking habits (never, current, or former smoker), alcohol consumption at follow-up, and quantity of physical activity measured using the QPAQ at follow-up. Profile 1 was used as the reference category. Belonging to a specific baseline profile (Figure 4A) did not determine BMI loss after surgery. On the other hand, we showed that Profile 3 represented an independent factor of BMI loss with a negative pattern (negative estimate; *p* = 0.0487) (Figure 4B).

### 3.2. Obesity-Related Complications (ORCs)

**Figure 5:**
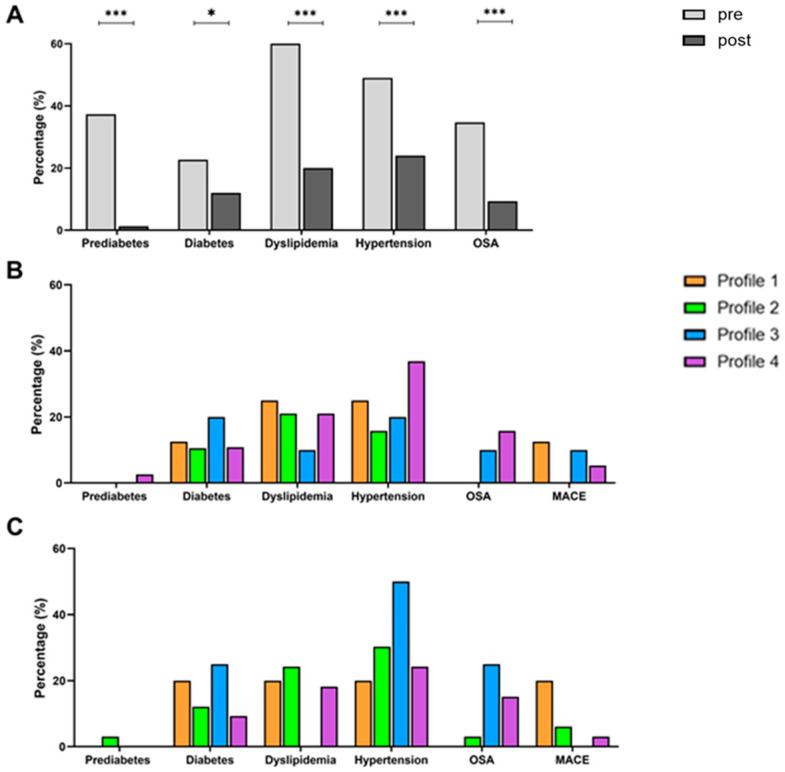
Prevalence of ORCs in percentages before and after bariatric surgery (**A**) for each baseline profile (**B**) and follow-up profile (**C**). *: *p* < 0.05; ***: *p* < 0.001. Profile 1: meal eaters; Profile 2: meal and snack eaters; Profile 3: continuous day and night eaters; Profile 4: continuous day eaters; OSA: Obstructive Sleep Apnea; MACE: Major Adverse Cardiovascular Event.

At baseline, 37.33% of the patients had prediabetes, 22.67% diabetes, 60% dyslipidemia, 49% hypertension, and 34.67% OSA (Table 1). After bariatric surgery, these values dropped to 1.3% for prediabetes (*p* < 0.0001), 12% for diabetes (*p* = 0.0233), 20% for dyslipidemia (*p* < 0.0001), 28% for hypertension (*p* < 0.001), and 9,3% for OSA (*p* < 0.001) (Table 1), displaying an overall significant remission of comorbidities after weight loss (Figure 5A).

The prevalence of ORCs for each profile, 3 years after bariatric surgery, considering the classification before surgery, is represented in Figure 5B and reported in Table 1. After surgery, only one patient with Profile 4 displayed prediabetes. Profile 2 (n = 2, 10.53%) and Profile 4 (10.81%) were the groups with the lowest prevalence of diabetes (*p* = 0.8995). Profile 3 represented the group with the lowest prevalence of dyslipidemia (n = 1, 10%). Profile 4 was the group with the highest prevalence of hypertension (n = 14, 36.84%, *p* = 0.3795). Profile 3 (n = 1, 10%) and Profile 4 (n = 6, 15.79%) presented the highest prevalence of OSA, but this was not significant (*p* = 0.2158). Profile 2 was the group with the lowest prevalence of diabetes (vs Profile 1, *p* = 1; vs. Profile 3, *p* = 0.592; vs. Profile 4, *p* = 1) and hypertension (vs. Profile 1, *p* = 0.616; vs. Profile 3, *p* = 1; vs. Profile 4, *p* = 0.132) and did not have any MACE. Profile 3 was the group with the lowest prevalence of dyslipidemia (vs Profile 1, *p* = 0.537; vs. Profile 2, *p* = 0.633; vs. Profile 4, *p* = 0.661). Profiles 1 and 2 did not suffer from OSA (Profile 1 vs. Profile 2, *p* = 1; Profile 1 vs. Profile 3, *p* = 1; Profile 1 vs. Profile 4, *p* = 0.571; Profile 2 vs. Profile 3, *p* = 0.345; Profile 2 vs. Profile 4, *p* = 0.164). Based on patients’ follow-up profiles, the prevalence of comorbidities is represented in Figure 5C and reported in Table 2. Nevertheless, despite some apparent differences found among baseline and follow-up profiles, no statistically significant differences in the prevalence of ORCs were found.

We found statistically significant differences in Epworth values, which are a marker of the prevalence of OSA, after bariatric surgery among the four follow-up profiles (*p* = 0.047), with the largest difference being between Profile 2 (three meals and two snacks) and Profile 4 (continuous day eaters) (*p* = 0.008) (Table 2). These differences were not observed when considering the baseline profiles. We did not find any differences in physical activity, quantified using the GPAQ, between the four baseline profiles (*p* = 0.2548) or between the four follow-up profiles (*p* = 0.72) (Table 1 and Table 2).

To determine whether belonging to a specific profile determines the remission or the persistence of one comorbidity, we performed a linear regression analysis only on the most frequent ORCs (hypertension, dyslipidemia, and diabetes), due to the low prevalence of the other ones (prediabetes and OSA). We used gender, age, smoking habits (never, current, or former smoker), alcohol consumption at follow-up, change in BMI, and quantity of physical activity measured via QPAQ at follow-up as predictors or confounding variables. According to the forest plots, only age has a statistically significant effect on both hypertension and dyslipidemia, considering both profiles at baseline (Table S1A) and follow-up (Table S1B). We did not identify any variables that influenced the presence of diabetes. Therefore, belonging to a particular profile did not influence the remission of hypertension, dyslipidemia, or diabetes.

## 4. Discussion

In the present study, we investigated whether belonging to a specific chrono-nutritional eating profile was associated with differences in weight loss and/or the likelihood of remission of ORCs three years after bariatric/metabolic surgery. We also investigated the potential movement of patients to a different nutritional profile after surgery, analyzing the association between the new nutritional profile acquired after surgery with both weight loss and ORC remission.

Although there was significant weight loss from baseline to follow-up across all 75 patients, no statistically significant differences in weight loss among the four chrono-nutritional profiles, considered at baseline or follow-up, were found in an unadjusted analysis. However, in a regression model including gender, age, presence of diabetes at baseline, smoking habits, alcohol consumption at follow-up, quantity of physical activity measured via the GPAQ at follow-up as covariates, only Profile 3 (“continuous day and night eaters”) was independent associated with a lower BMI loss compared to Profile 1 at follow-up. Although the percentage of patients in Profile 3 dropped from baseline to after surgery (from 10 to 4 patients), patients with this profile after surgery presented less weight loss compared to other profiles.

In our previous study, patients belonging to Profile 3 presented a major impairment in their psychological status [28] compared to Profile 1 and Profile 4 (“continuous day eaters”), highlighting a relationship between psychological issues and chrono-nutritional patterns [28]. A bidirectional association between night eating and mental health, which can influence the quality of food intake, has been demonstrated [34,35]. This association seems to be more related to nocturnal behaviour itself rather than overeating in general or increases in BMI. In our population, no significant differences in the baseline BMI or BMI loss between Profile 3 and Profile 4 were found. The association of Profile 3 with a lower BMI loss confirms a link between a disruption of eating habits throughout the day and overall food intake [15]. Moreover, an evening chronotype is associated with irregular eating and meal skipping, and a higher intake of energy drinks and fat [16,17]. It was also demonstrated that the evening chronotype is correlated with both metabolic disease and psychiatric symptoms [18] and an increased risk of all-cause mortality [19]. Conversely, a study conducted with over fifty thousand participants in the US and Canada showed that subjects who consumed their largest meal at breakfast rather than dinner had a significant decrease in their BMI in a 7-year follow-up [20]. The same finding was demonstrated in healthy individuals [22], and several studies confirmed the relationship between the time of eating, weight management, and the presence of comorbidities in subjects with overweight and obesity [21,23,24].

Temporal restriction of food intake has been considered as a determining factor for maintaining weight and the treatment of ORCs. In a short-term trial, 11 overweight adults were randomized to either 4 days of TRE or a control schedule. In the TRE group, levels of the satiety hormone peptide YY and subjective “stomach fullness” were higher, and ghrelin, perceived “hunger”, and the “desire to eat” were all significantly reduced. Metabolic flexibility was also increased in the TRE group, which burned fat more effectively during their fasting period [27]. Gill et al. developed a smartphone app to monitor food intake in healthy adults, revealing frequent and erratic daily eating patterns rather than the self-reported three daily meals, leading to ‘‘metabolic jetlag’’ [36]. In a pilot study, overweight individuals who used the app to restrict food intake to only 10–11 hr/day experienced sustained weight loss, despite not receiving any recommendations on nutrition or calories.

In this study, the prevalence of the four profiles could have been affected by selection bias, given that patients were recruited only if they underwent bariatric surgery and attended the 3-year follow-up. While Profile 1 was the most prevalent in our original sample of 173 patients [28], Profile 4 was the most prevalent in our later sample of 75 patients. After weight loss, the main difference in the distribution of profiles was the increase in the patients belonging to Profile 2 (from 19 to 33 patients). In fact, it is expected that people after bariatric surgery face challenges with portion size because of both anatomical alterations and hormonal adjustments. To overcome these issues, patients are usually recommended to eat small meals and consume snacks, which may explain the high prevalence of patients in Profile 2 after surgery. Profile 4, associated with an eating irregular pattern and nibbling, was also more prevalent. In our study, we did not demonstrate a relationship between belonging to a specific chrono-nutritional profile and compliance, but our data emphasize that chrono-nutritional patterns are not a stable individual characteristic. Chrono-nutritional patterns frequently change after surgery (Figure 1), both in a positive direction, with several patients originally belonging to a disrupted pattern (Profiles 3 and 4) transitioning to a more regular pattern (Profile 2), but also in a negative way, with some patients with a previous regular pattern acquiring an irregular one. This observation stresses the importance of dietetic and psychological follow-up not only when preparing for surgery, but also in the years after the procedure.

Circadian rhythms and temporal eating patterns have been associated with metabolic health [7,8,18,19,24,25,26,37]. A prospective study of over 100,000 French participants revealed that each 1 h delay in the timing of the first meal was associated with a 6% higher risk of overall cardiovascular disease, and every 1 h increase in fasting during the night resulted in a 7% lower risk of cerebrovascular diseases [38]. In this context, we aimed to underline a possible relationship between belonging to a specific chrono-nutritional profile and the presence and remission of ORCs. We did not find any statistically significant differences in the prevalence of ORCs between profiles, even though we found an apparent higher prevalence of diabetes, hypertension (only at follow-up), and OSA (only at follow-up) in patients with Profile 3. On the other hand, we evidenced a trend suggesting that baseline Profile 2 was the group with the lowest prevalence of diabetes and hypertension, and no cases of OSA or MACE. Moreover, it seems that Profile 3 was affected by a reduction in the remission of ORCs after bariatric surgery, while Profile 2 was associated with a decrease in the prevalence of ORCs. Consensually, an evening chronotype has been associated with poorer glycemic control in patients with diabetes and an increased risk of all-cause mortality over 6.5 years [8,17,18,19,24]. Furthermore, TRE has been shown to decrease mean 24 h glucose and glycemic excursions [27], blood pressure, and oxidative stress even without weight loss [39].

As a marker of the prevalence of OSA, we found a significant difference in Epworth scores after bariatric surgery among the four follow-up profiles (*p* = 0.047), with the largest difference observed between Profile 2 and Profile 4. The median Epworth score was 3 for Profile 2 (1–6) and 5 for Profile 4 (4–9); both scores are within the range of normal daytime sleepiness, with Profile 4 being closer to the limit for average daytime sleepiness [31]. This finding aligns with previous research suggesting that irregular eating schedules and high meal frequency, particularly late meals, can lead to disrupted sleep and increased daytime sleepiness [40].

Our study has several limitations. Firstly, the small sample size, particularly at follow-up, limited comparison between groups, as Profiles 1 and 3 had only five and four patients, respectively, while most of the patients belonged to Profile 2 and Profile 4 (n = 33). Thus, our finding that Profile 3 predicts lower weight loss should be confirmed with a larger sample size before drawing strong clinical implications. Moreover, the lack of statistically significant evidence in the regression model for the most frequent ORCs (hypertension, dyslipidemia, and diabetes) can also be explained by the small sample size. Finally, the use of 24 h dietary recall on eating habits relies on patients’ memory, which can be inaccurate.

## 5. Conclusions

The lower weight loss observed in patients belonging to Profile 3 after surgery, characterized by irregular eating patterns through the day and night, suggests that chrono-nutrition may play a role in weight regulation. Due to the small sample size, we do not propose clear clinical implications; however, this study highlights the importance of chrono-nutritional profiles in patients with severe obesity following bariatric surgery. Our data emphasize that chrono-nutritional patterns are not a stable individual characteristic after weight loss. Nutritional assessments based on temporal eating patterns could also be included in medical evaluations before and after bariatric surgery. This assessment does not evaluate caloric or nutritional intake (which are important aspects of patients’ eating patterns) but instead clusters individuals based on the probability of food intake over a 24-h period. Thus, it could be recommended to receive strict dietary counselling before and after bariatric surgery to avoid unhealthy eating behaviour. However, further studies are needed to confirm our results and determine a causal connection between eating profiles, weight loss, and remission of ORCs after bariatric surgery.

## Figures and Tables

**Table 1:** Characteristics of the population considering the baseline profile before (Pre) and after surgery (Post).

	Overall (n = 75)		Profile 1 (n = 8)		Profile 2 (n = 19)		Profile 3 (n = 10)		Profile 4 (n = 38)		*p*	
	Pre	Post	Pre	Post	Pre	Post	Pre	Post	Pre	Post	Pre	Post
Age (years)	50.92 ± 11.57		51.62 ± 11.26		51.79 ± 9.56		48.1 ± 8.4		51.08 ± 13.39		0.8691	
Gender (F)	74.67%		62.5%		68.42%		70%		81.58%		0.5589	
Weight (kg)	114 (104.5–131.5)	81 (73–93)	121.5 (113–135.5)	90 (79.75–99.25)	115 (100–131.5)	80 (73–85)	112.5 (104.3–134)	76 (66.5–83.5)	113 (106–124.3)	80.5 (72.25–96.75)	0.7287	0.2337
Weight Loss %	27.6 (22.88–35.81)		27.2 (24.28–30.56)		28.7 (26.68–38.67)		34.2 (28.04–38.62)		26.3 (20.76–32.5)		0.07	
BMI (kg/m^2^)	42.2 (39.10–46.35)	29.5 (27.45–33.92)	43.5 (39.48–53.73)	33.2 (29.97–36.72)	41.1 (39.1–47.4)	29.04 (27.3–31.26)	42.9 (39.73–45.95)	27.7 (24.68–30.8)	42.3 (39.1–44.98)	29.7 (28.05–35.56)	0.783	0.117
BMI Loss %	27.6 (22.98–35.83)		27.2 (24.33–30.57)		28.7 (26.67–38.63)		34.2 (28.07–38.64)		26.3 (20.71–32.54)		0.1204	
Hypertension	37 (49%)	21 (28%)	7 (87.5%)	2 (25%)	8 (42.1%)	3 (15.79%)	3 (30%)	2 (20%)	19 (50%)	14 (36.84%)	0.0879	0.3795
OSA	26 (34.67%)	7 (9.3%)	3 (37.5%)	0 (0%)	6 (31.58%)	0 (0%)	4 (40%)	1 (10%)	13 (34.21%)	6 (15.79%)	0.9819	0.2158
Prediabetes	28 (37.33%)	1 (1.3%)	2 (25%)	0 (0%)	5 (26.32%)	0 (0%)	3 (30%)	0 (0%)	18 (47.37%)	1 (2.63%)	0.3482	1
Diabetes	17 (22.67%)	9 (12%)	3 (37.5%)	1 (12.5%)	4 (21.05%)	2 (10.53%)	2 (20%)	2 (20%)	8 (21.05%)	4 (10.81%)	0.7716	0.8995
Dyslipidemia	45 (60%)	15 (20.27%)	7 (87.5%)	2 (25%)	8 (42.11%)	4 (21.05%)	9 (90%)	1 (10%)	21 (55.26%)	8 (21.05%)	0.0656	0.8421
GPAQ (MET min/week)		960 (160–2160)		1760 (720–4575)		960 (340–3960)		1300 (550–1650)		840 (0–1620)		0.2548
Epworth		5 (2–8)		5.5 (2–8.25)		4 (2–5)		6.5 (2.5–9.75)		5 (2–8)		0.4018
MACE		4 (5.33%)		1 (12.5%)		0 (0%)		1 (10%)		2 (5.26%)		0.5236

Data are represented as mean ± SD, median (IQR), or frequency (%). Profile 1: meal eaters; Profile 2: meal and snack eaters; Profile 3: continuous day and night eaters; Profile 4: continuous day eaters; F: female; BMI: body mass index; OSA: Obstructive Sleep Apnea; GPAQ: Global Physical Activity Questionnaire; MACE: Major Adverse Cardiovascular Event.

**Table 2:** Characteristics of the population considering the follow-up profile before (Pre) and after surgery (Post).

	Overall (n = 75)		Profile 1 (n = 5)		Profile 2 (n = 33)		Profile 3 (n = 4)		Profile 4 (n = 33)		*p*	
	Pre	Post	Pre	Post	Pre	Post	Pre	Post	Pre	Post	Pre	Post
Age (years)	50.92 ± 11.57		53.4 ± 10.16		50.70 ± 12.33		50 ± 16.31		50.88 ± 10.88		0.9675	
Gender (F)	74.67%		20%		75.76%		50%		84.85%		0.0112 *	
Weight (kg)	114 (104.5–131.5)	81 (73–93)	120 (116–147)	85 (81–92)	111.5 (100–132.5)	80 (73–93)	127 (109–145)	100 (88–110.25)	115 (105–130)	80 (72–85)	0.3804	0.3836
Weight Loss %	27.6 (22.88–35.81)		33.3 (26.72–38.67)		26.9 (21.99–30.57)		24.3 (17.95–27.74)		31.8 (24.18–38.20)		0.1507	
BMI (kg/m^2^)	42.2 (39.10–46.35)	29.5 (27.45–33.92)	47.3 (45.9–47.50)	30.5 (29.04–33.62)	41.3 (37.9–46.6)	29.4 (27.82–33.76)	44.6 (41.58–48.62)	37.2 (33.60–39.17)	42.2 (39.3–43.4)	29.4 (27.06–33.51)	0.1782	0.389
BMI Loss %	27.6 (22.98–35.83)		33.4 (26.75–38.61)		26.9 (22.03–30.50)		24.3 (17.96–27.76)		31.8 (24.14–38.26)		0.2093	
Hypertension	37 (49%)	21 (28%)	3 (60%)	1 (20%)	14 (42.42%)	10 (30.3%)	2 (50%)	2 (50%)	18 (54.54%)	8 (24.24%)	0.8492	0.7826
OSA	26 (34.67%)	7 (9.3%)	2 (40%)	0 (0%)	9 (27.27%)	1 (3%)	2 (50%)	1 (25%)	13 (39.39%)	5 (15.15%)	0.7016	0.1932
Prediabetes	28 (37.33%)	1 (1.3%)	2 (40%)	0 (0%)	9 (27.27%)	1 (3%)	2 (50%)	0 (0%)	15 (45.45%)	0 (0%)	0.4974	1
Diabetes	17 (22.67%)	9 (12%)	1 (20%)	1 (20%)	9 (27.27%)	4 (12.12%)	1 (25%)	1 (25%)	6 (18.18%)	3 (9.3%)	0.92	0.9334
Dyslipidemia	45 (60%)	15 (20.27%)	4 (80%)	1 (20%)	18 (54.55%)	8 (24.24%)	2 (50%)	0 (0%)	21 (63.64%)	6 (18.18%)	0.7842	0.7791
GPAQ (MET min/week)		960 (160–2160)		840 (840–960)		920 (0–2520)		200 (90–1050)		1200 (360–1680)		0.7228
Epworth		5 (2–8)		4 (2–5)		3 (1–6)		4 (3.75–5)		5 (4–9)		0.047 *
MACE		4 (5.33%)		1 (20%)		2 (6.06%)		0 (0%)		1 (3.03%)		0.4745

Data are represented as mean ± SD, median (IQR), or frequency (%). *: *p* < 0.05. Profile 1: meal eaters; Profile 2: meal and snack eaters; Profile 3: continuous day and night eaters; Profile 4: continuous day eaters; F: female; BMI: body mass index; OSA: Obstructive Sleep Apnea; GPAQ: Global Physical Activity Questionnaire; MACE: Major Adverse Cardiovascular Event.

## Data Availability

Data were extracted from electronic clinical documentation, and patient confidentiality was protected by assigning anonymous identification codes.

## Supplementary Materials

The following supporting information can be downloaded at: https://www.mdpi.com/article/10.3390/nu17172901/s1, Table S1: Odd Ratios (OR) and relative 95% Confidence Interval (95%CI) for the presence of obesity-related complications hypertension, dyslipidemia and diabetes, considering baseline profiles (**A**) or follow-up profiles (**B**), respectively, estimated by a Logistic regression, with respect to the reference category: Gender [Female], Alcohol consumption [No], Smoking habit [No], Profile 1.
