# Supplementary material for: Association of Chrono-Nutritional Profiles with Weight Loss and Comorbidity Remission After Bariatric Surgery in Patients with Severe Obesity
Source: Nutrients. 2025 Sep 8;17(17):2901. doi: 10.3390/nu17172901 (PMC12430155; doi:10.3390/nu17172901)
Supplement: Supplementary file 1 [file nutrients-17-02901-s001.zip › nutrients-3760079-SI.pdf]

Table S1 A-B. Odd Ratios (OR) and relative 95% Confidence Interval (95%CI) for the presence of obesity-related complications hypertension, dyslipidemia and diabetes, considering baseline profiles (**A**) or follow-up profiles (**B**), respectively, estimated by a Logistic regression, with respect to the reference category: Gender [Female], Alcohol consumption [No], Smoking habit [No], Profile 1. The bold refers to the statistically significant value. Profile 1: meal eaters, Profile 2: meal and snack eaters, Profile 3: continuous day and night eaters, Profile 4: continuous day eaters. Alcohol cons.: alcohol consumption, BMI: body mass index, GPAQ: Global Physical Activity Questionnaire.

A

Predictors	**Hypertension**			**Dyslipidemia**			**Diabetes**		
	OR	*95%CI*	*p*	*OR*	*95%CI*	*p*	*OR*	*95%CI*	*p*
(Intercept)	0.001	0.00-0.16	0.007	0.001	0-0.14	0.008	0.045	0.00-5.19	0.2
Gender	2.856	0.68-12.09	0.154	0.794	0.17-3.78	0.772	3.539	0.73-17.22	0.118
Age	1.114	1.04-1.2	**0.003**	1.14	1.95-1.24	**0.003**	1.02	0.95-1.09	0.567
Never smoked	0.438	0.07-2.78	0.382	0.693	0.08-6.14	0.742	3.478	0.192-63.14	0.399
Current smoker	0.621	0.06-6.28	0.686	0.708	0.05-9.91	0.798	6.64	0.26-169.52	0.252
Former smoker	2.742	0.16-46.78	0.486	1.648	0.09-31.09	0.739	3.833	0.09-167.54	0.486
Alcohol cons. [current]	0.236	0.03-1.63	0.143	0.041	0.002-0.95	0.047	0.129	0.01-2.18	0.486
BMI difference	0.997	0.88-1.13	0.961	1.033	0.91-1.17	0.609	0.979	0.87-1.11	0.738
GPAQ at follow up	1	1-1	0.847	1	1-1	0.593	1	0.99-1	0.132
Profile 2 at baseline	0.859	0.09-8.29	0.896	0.74	0.06-8.55	0.809	1.37	0.12-16.24	0.803
Profile 3 at baseline	1.43	0.09-22.72	0.8	0.352	0.02-7.86	0.51	1.94	0.13-29.3	0.632
Profile 4 at baseline	2.568	0.28-23.28	0.402	0.467	0.04-4.9	0.525	0.635	0.07-5.98	0.691

B

Predictors	**Hypertension**			**Dyslipidemia**			**Diabetes**		
	OR	*95%CI*	*p*	*OR*	*95%CI*	*p*	*OR*	*95%CI*	*p*
(Intercept)	0.001	0.00-0.14	0.006	0.001	0-0.304	0.018	0.103	0.00-11.76	0.347
Gender	3.07	0.61-15.43	0.173	0.735	0.13-4.15	0.728	3.403	0.58-20.16	0.177
Age	1.114	1.04-1.2	**0.002**	1.146	1.05-1.25	**0.003**	1.016	0.95-1.08	0.627
Never smoked	0.384	0.06-2.33	0.298	0.906	0.09-8.98	0.933	2.933	0.18-47.93	0.45
Current smoker	0.667	0.07-6.18	0.721	0.88	0.06-13.65	0.927	3.926	0.19-80.87	0.376
Former smoker	1.275	0.09-18.24	0.86	4.075	0.17-95.56	0.383	3.297	0.08-129.99	0.525
Alcohol cons. [current]	0.233	0.03-1.58	0.136	0.034	0.001-0.85	0.039	0.142	0.01-2.57	0.187
BMI difference	0.995	0.88-1.13	0.931	0.998	0.88-1.14	0.979	1	0.88-1.13	0.989
GPAQ at follow up	1	1-1	0.512	1	1-1	0.511	1	0.99-1	0.130
Profile 2 at follow-up	2.981	0.193-46	0.434	0.278	0.01-7.55	0.447	0.477	0.03-7.68	0.601
Profile 3 at follow-up	2.782	0.08-103.64	0.579	0.054	0-5.95	0.224	0.585	0.02-20.92	0.769
Profile 4 at follow-up	2.508	0.16-39.43	0.513	0.241	0.01-6.16	0.39	0.43	0.02-7.77	0.567
